# The long-term efficacy and tolerability of oral deferasirox for patients with transfusion-dependent β-thalassemia in Taiwan

**DOI:** 10.1007/s00277-015-2476-y

**Published:** 2015-09-25

**Authors:** Hsiu-Hao Chang, Meng-Yao Lu, Steven Shinn-Forng Peng, Yung-Li Yang, Dong-Tsamn Lin, Shiann-Tarng Jou, Kai-Hsin Lin

**Affiliations:** Department of Pediatrics, National Taiwan University Hospital, National Taiwan University College of Medicine, Taipei, Taiwan; Department of Medical Imaging, National Taiwan University Hospital, National Taiwan University College of Medicine, Taipei, Taiwan; Department of Laboratory Medicine, National Taiwan University Hospital, National Taiwan University College of Medicine, Taipei, Taiwan; Division of Pediatric Hematology/Oncology, Department of Pediatrics, National Taiwan University Hospital, National Taiwan University College of Medicine, 8 Chung-Shan South Road, Taipei, 100 Taiwan

**Keywords:** β-thalassemia, Cardiac iron, Deferasirox, Iron overload

## Abstract

Deferasirox is a novel once-daily, oral iron chelator. The aim of this study was to evaluate the long-term efficacy and tolerability of deferasirox in Taiwanese patients with transfusion-dependent β-thalassemia who have been treated with deferasirox for 7 years. Taiwanese patients aged ≥2 years with transfusion-dependent β-thalassemia whose serum ferritin levels were ≥1000 ng/mL and had started deferasirox treatment since December 2005 at the National Taiwan University Hospital were enrolled. Sixty patients were recruited for analysis, and 11 (18.3 %) patients discontinued deferasirox during the study. In the 42 patients included in the efficacy analysis, the mean serum ferritin levels decreased significantly by 2566 ng/mL after 7 years of treatment (*P* < 0.001). Forty-one of these patients received a cardiac T2* evaluation after 3 years of deferasirox treatment, and the mean cardiac T2* value increased significantly from 30.6 ± 16.6 to 45.9 ± 22.6 ms after 7 years of deferasirox treatment (*P* < 0.001). Deferasirox-related adverse events assessed by investigators were reported in 46 (76.7 %) patients. The most common adverse events related to deferasirox were skin rashes (*n* = 29, 48.3 %), followed by abdominal pain (*n* = 23, 38.3 %) and diarrhea (*n* = 16, 26.7 %). Most adverse events were manageable. This study demonstrated that long-term treatment with deferasirox was effective in improving iron overload, including cardiac iron overload, in patients with transfusion-dependent β-thalassemia. Deferasirox was well tolerated; however, the incidences of common adverse events related to deferasirox appeared higher in our Taiwanese patients than other studies.

**Electronic supplementary material:**

The online version of this article (doi:10.1007/s00277-015-2476-y) contains supplementary material, which is available to authorized users.

## Introduction

Thalassemia is a hereditary chronic anemia disorder. It is highly prevalent in the broad geographic belt that extends along the shores of the Mediterranean and throughout the Arabian peninsula, Turkey, Iran, India, and southeastern Asia, including Taiwan [1]. β-thalassemia is an important form of thalassemia and is derived from a defect in β-globin chain production [2]. In Taiwan’s population of over 23 million people, approximately 1.1 % are carriers for β-thalassemia [3]. Transfusion-dependent β-thalassemia, also known as β-thalassemia major, most often results from homozygosity or compound heterozygosity of a mutant β-thalassemia allele. Patients with transfusion-dependent β-thalassemia usually develop severe life-threatening anemia, hepatosplenomegaly, growth retardation, jaundice, and bone changes within the first year of life [4, 5]. These patients require regular red blood cell transfusions to save their lives; however, regular transfusions lead to iron overload [6]. Repeated red blood cell transfusions result in iron deposition in various organs and tissues, primarily the liver, heart, and the endocrine glands, thus causing tissue damage, leading to organ dysfunction and failure [2]. Therefore, iron overload causes significant morbidity and mortality in patients with transfusion-dependent β-thalassemia [2].

Previous studies have established the role of the iron chelator deferoxamine in preventing complications related to iron overload and improving overall survival of patients with transfusion-dependent β-thalassemia [7, 8]. Unfortunately, administration of deferoxamine by parenteral infusion results in poor compliance of patients and thus limits its efficacy in long-term iron chelation. Deferasirox (Exjade^®^) is a novel once-daily, oral iron chelator and represents a new class of tridentate iron chelator [9]. The efficacy and safety of deferasirox in patients with thalassemia has been previously documented [10–14], and deferasirox is currently approved in many countries for the treatment of patients with transfusion-dependent iron overload ≥2 years of age [9]. In Taiwan, patients with transfusion-dependent β-thalassemia in our hospital began to join clinical trials of deferasirox in 2005, and deferasirox was approved and became commercially available in 2007. Although the once-daily oral administration of deferasirox has led to high patient satisfaction and compliance, data on its long-term efficacy and safety in patients with transfusion-dependent β-thalassemia remains limited [15]. Therefore, the aim of this study was to evaluate the long-term efficacy and tolerability of deferasirox in patients with transfusion-dependent β-thalassemia who have been treated with deferasirox as iron-chelating therapy for 7 years in Taiwan.

## Methods

### Study design and patient enrollment

Patients aged ≥2 years with transfusion-dependent β-thalassemia whose serum ferritin levels were ≥1000 ng/mL started to receive deferasirox in December 2005 at the National Taiwan University Hospital. These patients received regular blood transfusions (10–15 ml packed erythrocytes/kg body weight) every 2–4 weeks in our thalassemia clinics to maintain a hemoglobin level of at least 9.5 g/dL before each transfusion. The starting dose of deferasirox was fixed at 20 mg/kg/day and then titrated according to the efficacy and adverse events. Patients who received deferasirox treatment for at least 7 years were enrolled in the efficacy analysis of deferasirox-mediated iron chelation. Patients who received at least one dose of deferasirox were included in the safety evaluation of deferasirox treatment. The clinical data of these patients were obtained retrospectively by medical records review, and this study was approved by the Institutional Review Board of the National Taiwan University Hospital.

### Patient assessment

Serum ferritin, liver enzymes, pancreatic enzymes, and serum creatinine levels were measured monthly in these patients, and any adverse events related to deferasirox were also recorded at every clinic visit. Cardiac iron load was assessed since 2009 at 1- or 2-year intervals by measuring myocardial T2* as previously described in patients ≥10 years of age [16]. The iron-chelating efficacy was evaluated by the change in serum ferritin levels and cardiac T2* values. The adverse events related to deferasirox in our patients were evaluated according to the Common Terminology Criteria for Adverse Events (US Department of Health and Human Services, National Institutes of Health, National Cancer Institute, Bethesda, MD, USA).

### Statistical analysis

Continuous variables were summarized by descriptive statistics, including the mean and range or standard deviation. Categorical variables were presented as the number and percentage in each category. The comparison of serum ferritin, cardiac T2*, and creatinine levels between the baseline value before deferasirox initiation and the last observed value was performed by a paired Wilcoxon signed-rank test. A statistically significant difference was defined as a *P* < 0.05. Statistical analyses were performed with SPSS 13.0 (SPSS Inc., Chicago, IL, USA).

## Results

### Patient characteristics and discontinuations

Sixty consecutive Taiwanese patients with transfusion-dependent β-thalassemia who had received deferasirox since December 2005 in our hospital were recruited for this study. The basic clinical characteristics of these 60 patients at the time that they started deferasirox treatment are listed in Table 1. Among these 60 patients, 11 (18.3 %) discontinued deferasirox during the follow-up period. Their reasons for discontinuation, alternative treatment of iron-chelating therapy, and outcomes are listed in Supplemental Table 1. Three (5 %) patients discontinued deferasirox because of adverse events. One patient died of *Yersinia* pneumonia after receiving deferasirox for 7 months, and one patient had reactivation of hepatitis C infection after 48 months of deferasirox treatment and therefore discontinued deferasirox. These two events were not related to deferasirox treatment. Another patient had bilateral ankle joint pain after receiving deferasirox for 60 months, which was considered to be related to deferasirox, and this patient discontinued deferasirox after 75 months of treatment. Additionally, four (6.7 %) patients discontinued deferasirox owing to personal concerns and most of these patients had compliance issues with deferasirox treatment.

**Table 1:** Basic demographic characteristics of the 60 Taiwanese patients with transfusion-dependent β-thalassemia at the initiation of deferasirox treatment

Characteristics	Total patients (*n* = 60)
Male, *n* (%)	27 (45)
Mean age (years) (range)	19.6 (2.6–34.4)
Age group, *n* (%)	
2 to <16 years	24 (40)
≥16 years	36 (60)
Serum ferritin category, *n* (%)	
≤2500 ng/mL	22 (37)
>2500–5000 ng/mL	18 (30)
≥5000 ng/mL	20 (33)

### Efficacy and deferasirox dosing

**Fig. 1:**
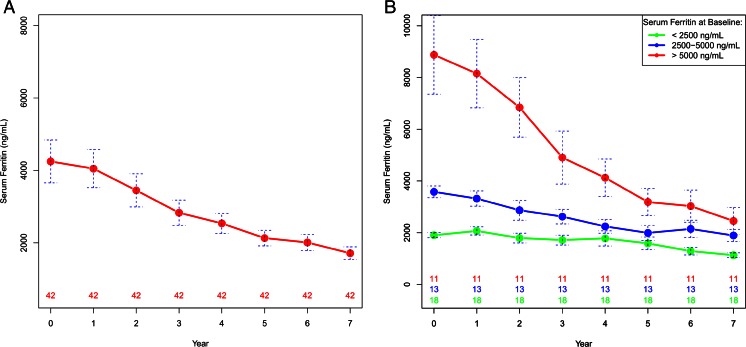
The distribution of serum ferritin levels in the 42 patients included in the efficacy analysis of deferasirox treatment. **a** The plot of mean serum ferritin levels at baseline (year 0) and over time with the standard error for all patients. **b** The group mean plot of serum ferritin levels at baseline (year 0) and over time with the standard error for patients grouped by different baseline serum ferritin levels. Patient numbers in the different groups are as indicated

The efficacy analysis was generated based on patients who received deferasirox treatment for at least 7 years. Of the 49 patients who continued deferasirox treatment, seven patients were excluded from the efficacy analysis because they moved to other cities during the study period and their data were incomplete. Therefore, a total of 42 patients were included in the efficacy analysis of iron chelation by deferasirox. The mean serum ferritin levels of these patients decreased significantly from 4279 ng/mL at the start of deferasirox treatment (baseline) to 1713 ng/mL after 7 years of deferasirox treatment (*P* < 0.001, Table 2). When we divided these patients into different groups according to their baseline serum ferritin levels (<2500 ng/mL, 2500 to 5000 ng/mL, >5000 ng/mL), the mean serum ferritin levels decreased significantly after 7 years of deferasirox treatment in all groups of patients (Table 2). The distribution of serum ferritin levels in these patients at baseline and after 7 years of deferasirox treatment is shown in Fig. 1. The relative change in serum ferritin from baseline over time is shown in Supplemental Fig. 1. A total of 11 (26 %) patients achieved serum ferritin levels below 1000 ng/mL after 7 years of deferasirox treatment (Table 3), which is the recommended safe serum ferritin level to maintain according to the guidelines of the Thalassemia International Federation [17].

**Table 2:** Comparisons of mean serum ferritin (SF) levels between baseline and after 7 years of deferasirox treatment in all patients and in patients with different baseline SF levels, who were included in the efficacy analysis of deferasirox

	All patients (*n* = 42)	Baseline SF groups (ng/mL)		
		<2500 (*n* = 18)	2500–5000 (*n* = 13)	>5000 (*n* = 11)
Mean (range) SF at baseline	4279 (1042–22,320)	1905 (1042–2464)	3609 (2546–4871)	8956 (5345–22,320)
Mean (range) SF after 7 years deferasirox	1713 (650–5842)	1130 (650–1902)	1892 (723–4091)	2454 (755–5842)
Mean (range) absolute change in SF	−2566 (−20,919–353)	−775 (−1647–239)	−1717 (−3751–353)	−6501 (−20,919 to −1644)
*P* value	<0.001	<0.001	0.003	0.003

**Table 3:** The distribution of serum ferritin levels in 42 patients included in the efficacy analysis of deferasirox at baseline and after 7 years of treatment

Serum ferritin (ng/mL), *n* = 42 (100 %)		Baseline					
		<1000, *n* = 0 (0 %)	1000–2000, *n* = 10 (24 %)	2000–3000, *n* = 12 (28 %)	3000–4000, *n* = 4 (10 %)	4000–5000, *n* = 5 (12 %)	>5000, *n* = 11 (26 %)
Year 7	<1000, *n* = 11 (26 %)		5	4		1	1
	1000–2000, *n* = 22 (52 %)		5	6	2	3	6
	2000–3000, *n* = 4 (10 %)			2	1	1	
	3000–4000, *n* = 1 (2.5 %)						1
	4000–5000, *n* = 3 (7 %)				1		2
	>5000, *n* = 1 (2.5 %)						1

**Fig. 2:**
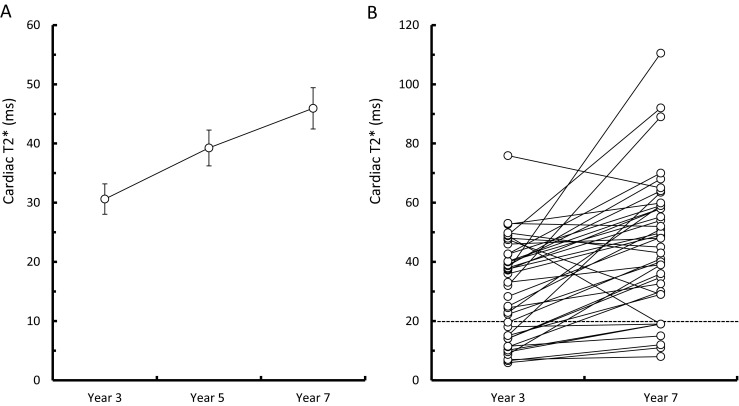
The distribution of cardiac T2* values of the 41 patients included in the efficacy analysis of deferasirox treatment who received myocardial iron load evaluation during the follow-up period. **a** The group mean plot of cardiac T2* with standard error after 3, 5, and 7 years of deferasirox treatment for all patients. **b** The plot of individual cardiac T2* values after 3 and 7 years of deferasirox treatment for all patients. The *dashed line* indicates a cardiac T2* value of 20 ms

Among the 42 patients included in the efficacy analysis, only one patient did not undergo cardiac T2* measurement in this study owing to his young age. In the remaining 41 patients, the mean cardiac T2* value increased significantly from 30.6 ± 16.6 ms after receiving deferasirox for 3 years (year 3) to 45.9 ± 22.6 ms after deferasirox treatment for 7 years (year 7) (*P* < 0.001). As cardiac T2* <20 ms is an indicator of abnormal myocardial iron content, the number of patients with cardiac T2* <20 ms decreased from 14 to 8 and only one patient’s cardiac T2* changed from >20 ms (48.8) to <20 ms (19) during this 4-year period. The mean cardiac T2* of these 14 patients increased significantly from 11.9 ± 4.2 to 26.9 ± 15.2 ms (*P* = 0.001) during this period. Half of these patients had baseline serum ferritin levels >5000 ng/mL, and eight of them had a mean deferasirox dose ≥30 mg/kg/day during the study period. Of the eight patients whose cardiac T2* values were still <20 ms after 7 years of deferasirox treatment, seven patients had improved cardiac T2* values when compared with the values at year 3. Their mean cardiac T2* increased significantly from 9.8 ± 4.2 ms at year 3 to 14.7 ± 4.5 ms at year 7 (*P* = 0.018). None of these eight patients had cardiac decompensation (LVEF ≤56 %) [18] after 7 years of deferasirox treatment. The distribution of cardiac T2* in patients after deferasirox treatment for 3 and 7 years is shown in Fig. 2.

The mean deferasirox dose of the patients included in the efficacy analysis during the 7 years of treatment was 27.4 ± 7.0 mg/kg/day. For patients with baseline serum ferritin levels of <2500, 2500–5000, and >5000 ng/mL, the mean deferasirox dose during the study was 23.2 ± 6.7, 29.5 ± 4.1, and 31.9 ± 6.3 mg/kg/day, respectively. The changes of deferasirox dosage in these patients over the study period are shown in Supplemental Fig. 2.

### Safety and tolerability

Deferasirox-related adverse events assessed by investigators were reported in 46 (76.7 %) patients during the study period. The most common adverse events were skin rashes (*n* = 29, 48.3 %), followed by abdominal pain (*n* = 23, 38.3 %) and diarrhea (*n* = 16, 26.7 %). Among the 29 patients who had rashes, three patients had two episodes and one patient had three episodes. Gastrointestinal adverse events related to deferasirox were reported in 29 (48.3 %) patients (Supplemental Table 2). Other deferasirox-related adverse events were elevated liver enzymes in five (8.3 %), ankle joint pain in three (5 %), and Fanconi syndrome in one (1.7 %) patients. Most of these drug-related adverse events were mild to moderate in severity, manageable, and happened in the first 2 years of deferasirox treatment.

**Fig. 3:**
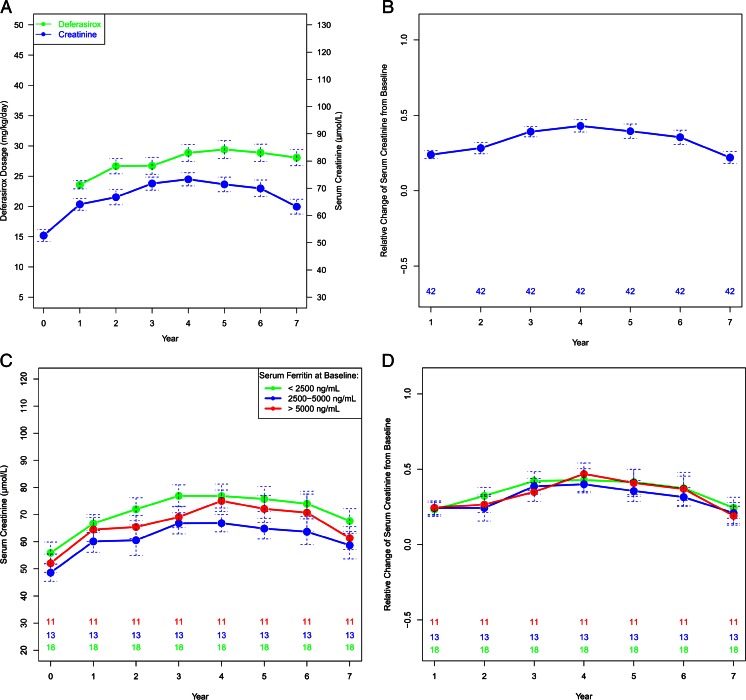
Serum creatinine levels of the 42 patients included in the efficacy analysis of deferasirox treatment over the study period. **a** The plot of mean serum creatinine levels versus deferasirox dosage at baseline (year 0) and over time with the standard error for all patients. **b** The plot of mean relative change of serum creatinine from baseline over time with standard error. **c** The group mean plot of serum creatinine levels at baseline and over time with standard error for patients grouped by different baseline serum ferritin levels. Patient numbers in the different groups are also shown. **d** The group mean plot of relative change of serum creatinine over time with standard error for patients grouped by different baseline serum ferritin levels

Four (6.7 %) patients had two consecutive elevated serum creatinine levels with more than a 33 % increase and greater than the upper normal limits. Their serum ferritin levels at the start of deferasirox were all above 3000 ng/mL, and they had taken a higher dosage of deferasirox (≥30 mg/kg/day) when their serum creatinine levels were abnormal. After adjusting deferasirox dosages, their serum creatinine levels returned to the normal range and they continued deferasirox treatment without any sequelae. The mean serum creatinine levels of the patients included in the efficacy analysis increased significantly from 52.6 μmol/L at baseline to 63.2 μmol/L after 7 years of deferasirox treatment (*P* < 0.001). The serum creatinine levels of these patients remained in the normal range after 7 years of deferasirox treatment. Their mean serum creatinine levels showed an increasing trend over time, which was parallel to deferasirox dosage (Fig. 3).

## Discussion

Although deferasirox has been available in many countries for many years, data on long-term efficacy and safety from patients with transfusion-dependent β-thalassemia are still limited. To the best of our knowledge, this report spans the longest observation period for evaluation of the efficacy and safety of deferasirox in patients with transfusion-dependent β-thalassemia. Thus, our study makes substantial contributions to the available long-term follow-up information of patients with transfusion-dependent β-thalassemia using deferasirox monotherapy. Our study recruited ethnically homogenous Asian patients, different from other studies [18, 19], providing more specific information for Asian patients when using deferasirox as an iron chelating agent.

Seven years of deferasirox treatment led to a sustained reduction in iron burden in most of our patients. In this study, we enrolled patients with transfusion-dependent β-thalassemia whose serum ferritin levels were ≥1000 ng/mL to receive deferasirox. The number of our patients whose serum ferritin levels were <2500 ng/mL, a level shown to reduce the risk of cardiac complications [7], increased from 18 (42.9 %) at baseline to 36 (85.7 %) after 7 years of deferasirox treatment. However, there were four patients whose baseline serum ferritin levels were >2000 ng/mL and their serum ferritin levels did not decrease more than 1000 ng/mL after 7 years of deferasirox treatment (Table 3). These patients may be unresponsive to once-daily deferasirox treatment, and alternative iron-chelating therapies should be considered for this kind of patient [20]. In addition, recent studies have shown that deferasirox monotherapy can significantly reduce myocardial iron content [21, 19, 18]. The changes of cardiac T2* in our patients which were described in the results and shown in Fig. 2 also demonstrated this effect over a 4-year period. For the ten patients who discontinued deferasirox and had been followed-up for 7 years in our study, the mean serum ferritin levels decreased from 5190 ± 3111 ng/mL at baseline to 3193 ± 2002 ng/mL after 7 years (*P* = 0.114, Supplemental Table 1). Their mean cardiac T2* increased from 15.1 ± 12.6 ms at year 3 to 21.7 ± 14 ms at year 7 during a 4-year period (*P* = 0.074, Supplemental Table 1).

In this study, we confirmed that deferasirox could reduce serum ferritin levels in a dose-dependent manner [15]. Our patients with transfusion-dependent β-thalassemia started deferasirox at a fixed dose of 20 mg/kg/day, which was then titrated according to efficacy and tolerability. After 7 years of treatment, serum ferritin levels decreased most in patients whose baseline serum ferritin levels were >5000 ng/mL (Table 2 and Supplemental Fig. 1). In this group of patients, there were nine (82 %, 9/11) patients whose mean deferasirox dose at the seventh year of treatment was ≥30 mg/kg/day. In contrast, patients whose baseline serum ferritin levels were <2500 ng/mL decreased their serum ferritin levels by 775 ng/mL (Table 2) and 37.3 % (Supplemental Fig. 1) after 7 years of deferasirox treatment. Only four (22 %, 4/18) patients out of those taking deferasirox at a mean dose ≥30 mg/kg/day remained at the seventh year of treatment.

For the safety profile, deferasirox was well tolerated over the long-term for all our patients in this study. Only one patient discontinued deferasirox treatment because of an adverse event related to deferasirox. The proportion of our patients reported to have deferasirox-related adverse events was similar to other studies [22]. The most common adverse events with a suspected relationship to deferasirox were also similar to other studies [15, 22, 23]. However, the incidences of common adverse events related to deferasirox were higher in our patients when compared with those in other studies. The frequency of deferasirox-related skin rashes in our patients was 48.3 %, which was reported in only 11.8 % of Chinese patients with transfusion-dependent β-thalassemia in a recent study [23]. Although our study had a longer follow-up period, the majority of skin rashes developed within 2 weeks (median was 11 days) of starting deferasirox treatment. Almost all of our patients developed these skin rashes within the first year of deferasirox treatment, and only one patient developed these after a little more than 1 year (404 days) of deferasirox treatment initiation. The exact reasons for the higher frequency of deferasirox-related skin rashes in our patients remain unclear. The differential frequency of deferasirox-related skin rashes in different geographic areas of patients with transfusion-dependent β-thalassemia has been noted in a previous study [22], and different genetic backgrounds related to ethnicity, clinical manifestations, or type of drug may be possible explanations for this observation [22]. Increased levels of liver enzymes and serum creatinine related to deferasirox were also noted in our study; however, the incidences of these two events were not higher when compared with other studies [23, 22, 15]. Fanconi syndrome was observed in one (1.7 %) patient in this study. This patient had lower serum ferritin levels (<1000 ng/mL) and infection episodes (cholangitis) when this event happened.

The limitations of this study included the retrospective analysis and the limited number of patients. Additionally, we did not have liver iron concentration (LIC) data to show the efficacy of deferasirox in reducing the iron burden specifically in the liver in this study. The tools to measure hepatic iron overload by liver MRI have only recently become available in our hospital. A study to follow up the long-term effects of deferasirox treatment specifically on hepatic iron overload in our patients with transfusion-dependent β-thalassemia is currently ongoing.

In conclusion, the results of this study demonstrated that long-term treatment with deferasirox was effective in improving iron overload, including cardiac iron overload, in most of our Taiwanese patients with transfusion-dependent β-thalassemia. A small number of our patients may be unresponsive to once-daily deferasirox, and an alternative iron-chelating therapy may be considered for this kind of patient. Long-term treatment with deferasirox was also well tolerated in our patients, but the incidences of common adverse events related to deferasirox appear higher in our Taiwanese patients compared with previous studies. Thus, the safety profile of deferasirox may be quite different between different geographic areas and ethnic groups. Most of these adverse events were manageable, but the long-term safety profile of deferasirox in our patients should be continuously monitored.

## Electronic supplementary material

Supplementary Table 1 (DOCX 18 kb)

Supplementary Table 2 (DOCX 16 kb)

Supplementary Fig. 1 (DOCX 101 kb)

Supplementary Fig. 2 (DOCX 99 kb)
