# Supplementary material for: The long-term efficacy and tolerability of oral deferasirox for patients with transfusion-dependent β-thalassemia in Taiwan
Source: Ann Hematol. 2015 Sep 25;94(12):1945–52. doi: 10.1007/s00277-015-2476-y (PMC4604499; doi:10.1007/s00277-015-2476-y)
Supplement: Supplementary file 1 — (DOCX 18 kb) [file 277_2015_2476_MOESM1_ESM.docx]

**Table S1** Reasons for patient discontinuation of deferasirox and their outcomes.

| Subject No. | Reason | Duration of deferasirox treatment | Alternative  treatment | SF at baseline | SF at discontinuation of deferasirox | SF at the time of analysis | Cardiac T2* (ms) at year 3 | Cardiac T2* (ms) at year 7 | Outcome |
| --- | --- | --- | --- | --- | --- | --- | --- | --- | --- |
| 1 | Unsatisfactory therapeutic effects | 18 months | deferiprone combined with deferoxamine | 8063 | 5337 | 879 | 4.8 | 33 |  |
| 2 | Unsatisfactory therapeutic effects | 28 months | deferiprone combined with deferoxamine | 4584 | 4961 | 1604 | 5.7 | 8.5 |  |
| 3 | Unsatisfactory therapeutic effects | 33 months | deferiprone combined with deferoxamine | 9729 | 16220 | 6095 | 5.5 | 8.1 |  |
| 4 | Unsatisfactory therapeutic effects | 36 months | deferiprone | 1075 | 2842 | 1590 | 11.2 | 10 |  |
| 5 | Pneumonia | 7 months | None | 3272 | 3096 | 3096 | ND | ND | Died of *Yersinia* pneumonia |
| 6 | Hepatitis C reactivation | 48 months | deferoxamine | 1864 | 1758 | 2335 | 43.3 | 47 | Hepatitis C controlled after antiviral treatment |
| 7 | Bilateral ankle joint pain | 75 months | deferiprone | 3146 | 2484 | 2633 | 24.2 | 40 | Joint pain not improved |
| 8 | Personal concerns | 11 months | deferiprone combined with deferoxamine | 8680 | 8086 | 2526 | 7.8 | 16 |  |
| 9 | Personal concerns | 12 months | deferiprone combined with deferoxamine | 5967 | 4502 | 2662 | 8.6 | 25 |  |
| 10 | Personal concerns | 24 months | deferiprone | 1977 | 3014 | 6747 | 27.4 | 11 |  |
| 11 | Personal concerns | 60 months | deferiprone | 6815 | 4372 | 4860 | 22 | 18 |  |

Abbreviations: SF, serum ferritin; ND, not determined.
