# Supplementary material for: The long-term efficacy and tolerability of oral deferasirox for patients with transfusion-dependent β-thalassemia in Taiwan
Source: Ann Hematol. 2015 Sep 25;94(12):1945–52. doi: 10.1007/s00277-015-2476-y (PMC4604499; doi:10.1007/s00277-015-2476-y)
Supplement: Supplementary file 2 — (DOCX 16 kb) [file 277_2015_2476_MOESM2_ESM.docx]

**Table S2** Gastrointestinal adverse events related to deferasirox in our patients.

| Event | No. of cases (total = 60) | Rate |
| --- | --- | --- |
| Abdominal pain | 23 | 38.3% |
| Diarrhea | 16 | 26.7% |
| Nausea | 14 | 23.3% |
| Vomiting | 8 | 13.3% |
| Loose stool | 5 | 8.3% |
| Abdominal discomfort | 4 | 6.7% |
| Acute gastroenteritis | 2 | 3.3% |
| Constipation | 1 | 1.7% |
| Peptic ulcer | 1 | 1.7% |
| Gastric ulcer | 1 | 1.7% |
| Total G.I. cases | 29 | 48.3% |
