# Supplementary material for: The long-term efficacy and tolerability of oral deferasirox for patients with transfusion-dependent β-thalassemia in Taiwan
Source: Ann Hematol. 2015 Sep 25;94(12):1945–52. doi: 10.1007/s00277-015-2476-y (PMC4604499; doi:10.1007/s00277-015-2476-y)
Supplement: Supplementary file 3 — (DOCX 101 kb) [file 277_2015_2476_MOESM3_ESM.docx]

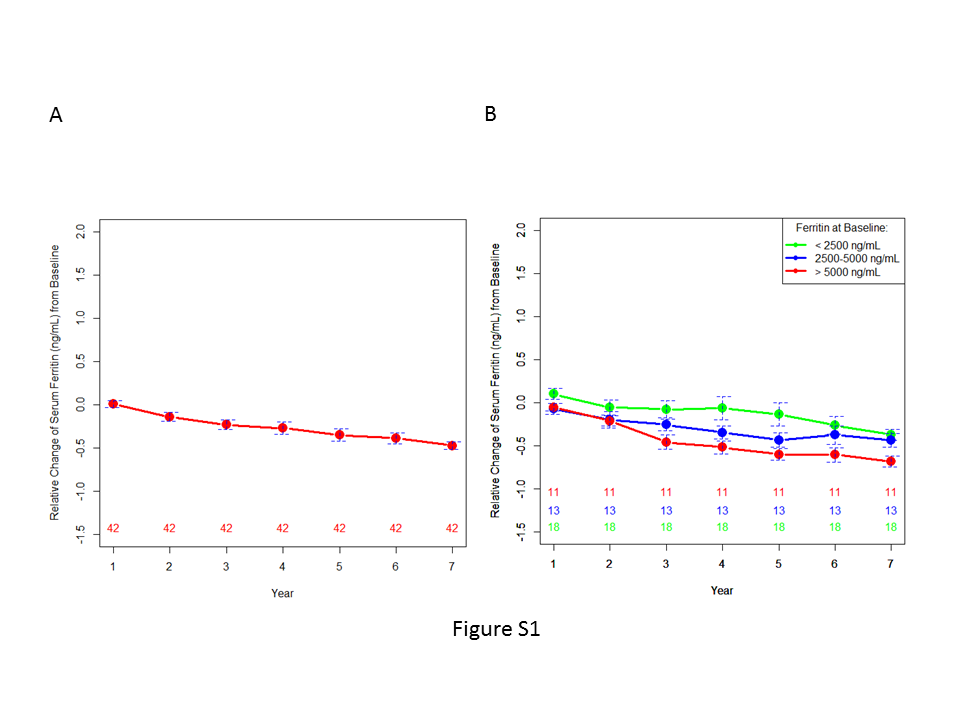


**Fig. S1** Relative changes in serum ferritin levels from baseline over time of the 42 patients included in the efficacy analysis of deferasirox treatment. (a) The mean plot of relative changes of serum ferritin from baseline over years with standard error for all patients. The mean relative change in serum ferritin from baseline for all patients over 7 years of deferasirox treatment was −47.5%. (b) The group mean plot of relative changes of serum ferritin from baseline over time with standard error for patients with different baseline serum ferritin levels. Patient numbers of the different groups are also shown (serum ferritin <2500, serum ferritin 2500–5000, serum ferritin >5000). For patients with baseline serum ferritin levels < 2500 ng/mL, 2500 –5000 ng/mL and > 5000 ng/mL, the mean relative changes in serum ferritin from baseline over 7 years of deferasirox treatment were −37.3%, −44%, and −68.3%, respectively.
