# Supplementary material for: The long-term efficacy and tolerability of oral deferasirox for patients with transfusion-dependent β-thalassemia in Taiwan
Source: Ann Hematol. 2015 Sep 25;94(12):1945–52. doi: 10.1007/s00277-015-2476-y (PMC4604499; doi:10.1007/s00277-015-2476-y)
Supplement: Supplementary file 4 — (DOCX 99 kb) [file 277_2015_2476_MOESM4_ESM.docx]

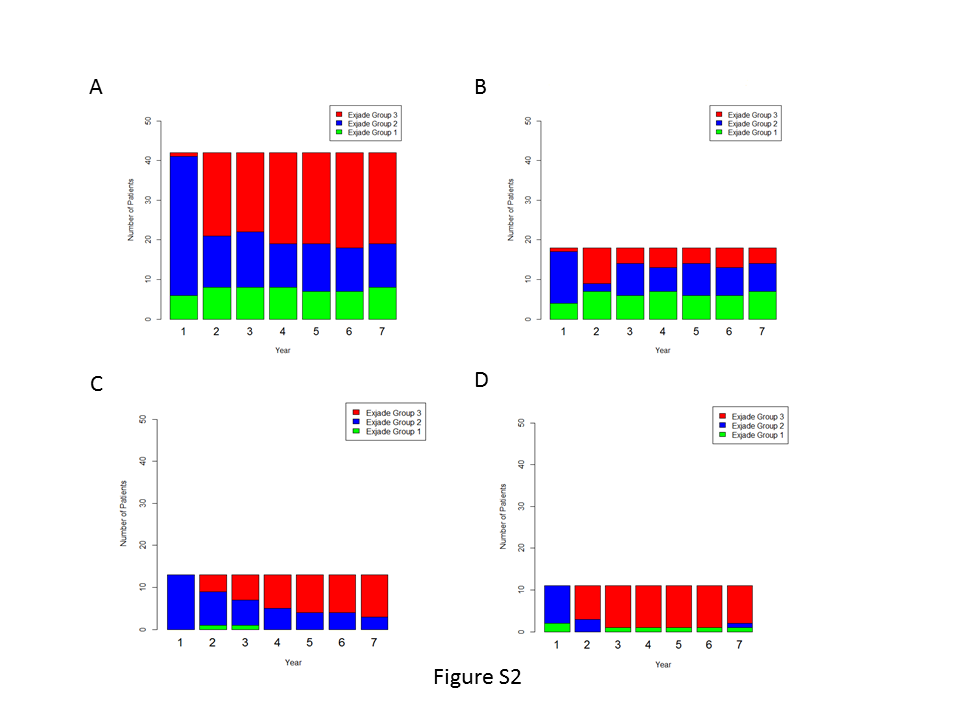


**Fig. S2** Changes in deferasirox dosages in patients included in efficacy analysis over the study period. (a) For all 42 patients. (b) For patients whose baseline serum ferritin levels were < 2500 ng/mL. (c) For patients whose baseline serum ferritin levels were 2500–5000 ng/mL. (d) For patients whose baseline serum ferritin levels were > 5000 ng/mL. Exjade (deferasirox) Group 1: < 20 mg/kg/day (green), Group 2: ≥ 20 and < 30 mg/kg/day (blue), Group 3: ≥ 30 mg/kg/day (red).
